# Supplementary material for: Conversion of Secondary C3-C4 Aliphatic Alcohols on Carbon Nanotubes Consolidated by Spark Plasma Sintering
Source: Nanomaterials (Basel). 2021 Feb 1;11(2):352. doi: 10.3390/nano11020352 (PMC7912505; doi:10.3390/nano11020352)
Supplement: Supplementary file 1 [file nanomaterials-11-00352-s001.pdf]

nanomaterials

Article

Conversion of Secondary C3-C4 Aliphatic Alcohols on Carbon Nanotubes Consolidated by Spark Plasma Sintering

Serguei Savilov [1,2], Evgeniya Suslova [1,*], Vsevolod Epishev [1], Evgeniya Tveritinova [1], Yuriy Zhitnev [1], Alexander Ulyanov [1], Konstantin Maslakov [1] and Oksana Isaikina [1]

[1] Department of Chemistry, Lomonosov Moscow State University, Leninskie Gory, Moscow 119991, Russia; savilov@chem.msu.ru (S.S.); olimpxumuk@gmail.com (V.E.); eatver@mail.ru (E.T.); zhitnev@kge.msu.ru (Y.Z.); a-ulyanov52@yandex.ru (A.U.); nonvitas@gmail.com (K.M.); hardi1@yandex.kz (O.I.)

[2] A.V. Topchiev Institute of Petrochemical Synthesis, Russian Academy of Sciences, Leninsky prospekt, 29, Moscow 119991, Russia

* Correspondence: suslova@kge.msu.ru

Abstract: We analyze how the changes in the dimension of carbon nanomaterial (CNM) affect their catalytic conversion of secondary aliphatic alcohols. Carbon nanotubes (CNTs) consolidated by spark plasma sintering (SPS) were inactive in the conversion of secondary C_3-C_4 aliphatic alcohols because of the «healing» of defects in carbon structure during SPS. Gas-phase treatment of consolidated CNTs with HNO_3 vapors led to their surface oxidation without destruction of the bulk structure of pellets. The oxygen content in consolidated CNTs determined by X-ray photoelectron spectroscopy increased from 11.3 to 14.9 at. % with increasing the oxidation time from 3 to 6 h. Despite the decrease in the specific surface area, the oxidized samples showed enhanced catalytic activity in alcohol conversion because of the increased number of oxygen radicals with unpaired electrons, which was established by electron paramagnetic resonance spectroscopy. We conclude that the structure of CNM determines the content and/or ratio of sp^2 and sp^3-hybridized carbon atoms in the material. The experimental and literature data demonstrated that sp^3-hybridized carbon atoms on the surface are probably the preferable site for catalytic conversion of alcohols.

Keywords: carbon nanotubes; spark plasma sintering; gas-phase oxidation; catalyst; dehydration; dehydrogenation; activation energy; secondary alcohols; electronic paramagnetic resonance

Citation: Savilov, S.; Suslova, E.; Epishev, V.; Tveritinova, E.; Zhitnev, Y.; Ulyanov, A.; Maslakov, K.; Isaikina, O. Conversion of Secondary C3-C4 Aliphatic Alcohols on Carbon Nanotubes Consolidated by Spark Plasma Sintering. *Nanomaterials* **2021**, *11*, 352. https://doi.org/10.3390/nano11020352

Academic Editor: Manuel J. Pérez-Mendoza

Received: 29 December 2020
Accepted: 23 January 2021
Published: date

Publisher's Note: MDPI stays neutral with regard to jurisdictional claims in published maps and institutional affiliations.

Copyright: © 2021 by the authors. Submitted for possible open access publication under the terms and conditions of the Creative Commons Attribution (CC BY) license (http://creativecommons.org/licenses/by/4.0/).

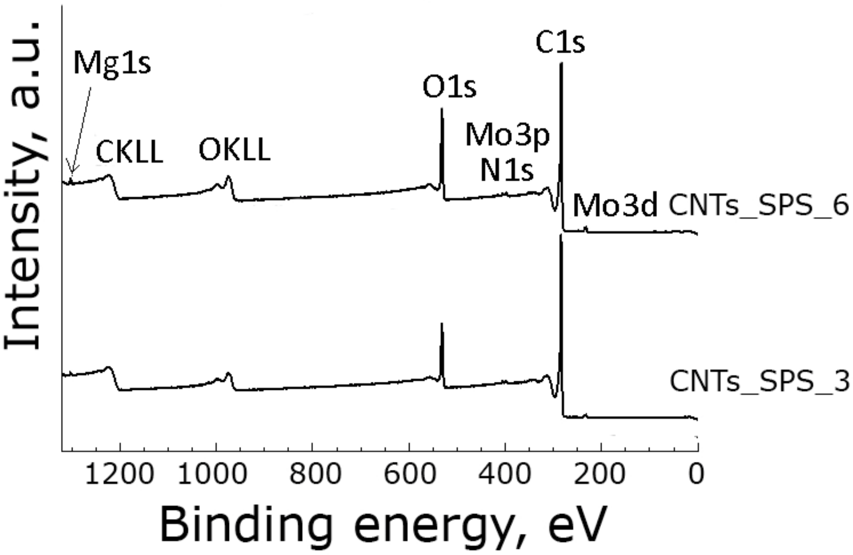

Figure S1. Survey XPS spectra of CNT samples.

Table S1. I_D/I_G ratios calculated from Raman spectra, total oxygen content and contents of carbon on oxygen species measured by XPS.

Sample	I_{D1}/I_G	I_{2D1}/I_G	Content, at. %					
			**Oxygen (Total)**	**O=C–O (531.0 eV)**	**H–O–C (532.0 eV)**	**O=C–O (533.1 eV)**	**C–C (*sp*³) (284.8 eV)**	**C–C (*sp*²) (284.3 eV)**
CNTs_raw	0.85	0.64	1.0					
CNTs_SPS	0.82	0.64	0.1					
CNTs_SPS_3	0.97	0.61	11.3	3.80	3.64	3.88	5.2	77.5
CNTs_SPS_6	0.99	0.60	14.9	5.52	4.25	5.14	7.6	70.2

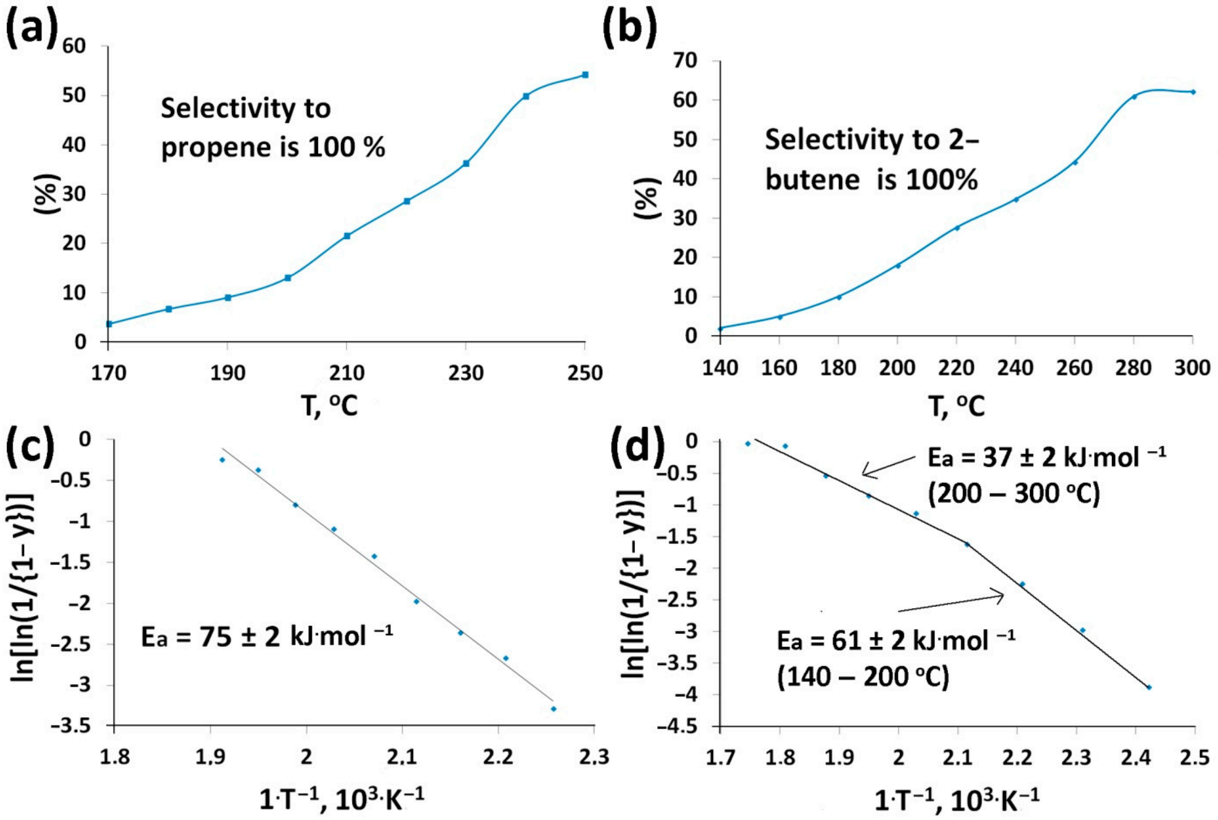

Figure S2. Temperature dependences of propanol-2 (**a**) and butanol-2 (**b**) conversions, and products selectivities over oxidized graphene nanoflakes. The activation energies of propanol-2 (**c**) and butanol-2 (**d**) conversion expressed in the Habgood-Bassett coordinates according to [36], where y is the fractional conversion.
